# Extracorporeal membrane oxygenation in non-intubated immunocompromised patients

**DOI:** 10.1186/s13054-021-03584-8

**Published:** 2021-04-30

**Authors:** Klaus Stahl, Heiko Schenk, Christian Kühn, Olaf Wiesner, Marius M. Hoeper, Sascha David

**Affiliations:** 1 Department of Gastroenterology, Hepatology and Endocrinology, Hannover Medical School, Hannover, Germany; 2 Department of Nephrology and Hypertension, Hannover Medical School, Hannover, Germany; 3 Department of Cardiothoracic, Transplant and Vascular Surgery, Hannover Medical School, Hannover, Germany; 4 Department of Respiratory Medicine and German Centre of Lung Research (DZL), Hannover Medical School, Hannover, Germany; 5 Institute of Intensive Care Medicine, University Hospital Zurich, Zürich, Switzerland

Veno-venous (VV) extracorporeal membrane oxygenation (ECMO) has become an integral part in the rescue therapy of severe acute respiratory distress syndrome (ARDS) and may be lifesaving in patients with refractory hypoxemia [1]. Ventilator-induced lung injury, ventilator-acquired pneumonia and ventilator-induced diaphragm dysfunction are severe side effects of invasive ventilation and may contribute to the complex pathophysiology of multi-organ failure and death in ARDS [2]. The use of ECMO in patients who are awake and spontaneously breathing (termed awake ECMO) might avoid side effects and complications associated with sedation, intubation and invasive mechanical ventilation [3]. Our group reported the first successful use of awake ECMO in six ARDS patients several years ago [4]. We then concluded that the concept of an awake ECMO strategy as a potential alternative to intubation deserves further evaluation especially in patients with higher mortality following traditional invasive ventilation and ECMO support.

In immunocompromised patients with ARDS who require ECMO support, the 6-month mortality exceeds 70% with a reported in-hospital mortality of 81% in patients following hematopoietic stem cell transplantation (HSCT) [5]. In immunocompromised patients with *Pneumocystis jirovecii*-associated pneumonia and severe ARDS we demonstrated earlier that a primarily awake ECMO strategy seems to be a promising strategy [6].

We therefore hypothesized that awake ECMO support to avoid invasive ventilation in selected immunocompromised patients might yield improved outcomes. Here, we present a comprehensive summary of 18 immunocompromised patients who received awake ECMO support for management of ARDS at our institution between 09/2012 and 09/2020.

The patient characteristics are shown in Table 1. At inclusion, the majority of patients had isolated lung failure indicated by rather low rates of low-dose vasopressor support (28%) and renal replacement therapy (6%) as well as physiological serum lactate concentrations. Median (Interquartile Range (IQR)) oxygenation indices at ECMO initiation were 72 (65–82) mmHg with normal values for pH and pCO_2_ despite maximal respiratory support by noninvasive ventilation (NIV). ECMO was initiated after a median of 1 (1–3) days following initial ICU admission and was carried out for 11 (9–18) days.

**Table 1:** Demographic and clinical characteristics at initiation of awake ECMO support

Characteristic at inclusion	Median (IQR)/no (%)
Number of patients	18
Age—years	54 (36–60)
Sex—no (%)	
Male	11 (61.1)
Female	7 (38.9)
BMI—kg/m^2^	24.5 (21.3–27.1)
Adipositas (BMI > 30 kg/m^2^)—no (%)	2 (11.1)
Cause of immunosupression—no (%)	
Solid organ transplant	7 (38.9)
HSCT	6 (33.3)
AIDS	2 (11.1)
Rheumatological disease	3 (16.7)
Pathogen—no (%)	
Gram +	2 (11.1)
Gram−	3 (16.7)
CMV	1 (5.6)
Fungal	2 (11.1)
PcP	8 (44.4)
Non-identified	5 (27.8)
Comorbidities—no (%)	
COPD	3 (16.7)
Cystic fibrosis	1 (5.6)
Pulmonary fibrosis	6 (33.3)
Pulmonary hypertension	1 (5.6)
Arterial hypertension	5 (27.8)
Congestive heart failure	1 (5.6)
Diabetes mellitus	3 (16.7)
Chronic kidney disease	5 (27.8)
Ventilation support—no (%)	
HFNC	1 (5.6)
NIV	17 (94.4)
Respiratory parameters	
FiO_2_	1 (1–1)
PEEP—cmH_2_O	7 (5–8)
Breaths per minute	32 (29–38)
Tidal volume—ml	593 (476–786)
Minute ventilation—l/min	21.3 (13.8–24.4)
Peak—cmH_2_O	17 (14–19)
P_a_O_2_—mmHg	65 (58–82)
PaO_2_/FiO_2_—mmHg	72 (65–82)
P_a_CO_2_—mmHg	40 (35–59)
pH	7.36 (7.3–7.44)
Lactate—mmol/l	1.5 (1.2–1.9)
Vasopressor—no (%)	5 (27.8)
Norepinephrine dose—μg/kg/min	0 (0–0.068)
Inotropic—no (%)	0 (0)
Renal replacement therapy—no (%)	1 (5.6)
SOFA-score (points)	7 (4–8)
Organ dysfunction—no (%)	
Respiratory (P_a_O_2_/FiO_2_ < 300)	18 (100)
Coagulation (thrombocytes < 100)	5 (27.8)
Liver (bilirubin > 33 μmol/l)	2 (11.1)
Cardiovascular (dobutamine or noradrenaline)	5 (27.8)
Neurological (GCS < 13)	0 (0)
Renal (creatinine > 170 μmol/l)	3 (16.7)
Multi-organ dysfunction—no (%)	
Two	11 (61.1)
Three	1 (5.6)
Four	1 (5.6)
Five	0 (0)
Six	0 (0)
CRP—mg/l	173 (78–226)
PCT—μg/l	0.8 (0.3–1.5)
Creatinine—μmol/l	69 (53–107)
24 h diuresis—ml	1230 (360–2165)
ECMO settings (directly after cannulation)	
Veno-venous	18 (100)
Cannulation (inflow–outflow)	
Femoral-jugular	16 (88.8)
Femoral–femoral	1 (5.6)
Femoral-subclavia	1 (5.6)
Pump speed—rpm	3410 (3030–3671)
Blood flow—l/min	3.6 (3.3–4)
FiO_2_	100 (100–100)
Sweep gas flow—l/min	3.5 (2–4.1)

Description of the patient cohort (n = 18) that received awake ECMO support. All immunocompromised patients with severe ARDS were non-systematically screened by two ECMO experienced ICU attending intensivists for the possibility of an awake ECMO strategy following inclusion and exclusion criteria that have been previously defined by our group as part of the study describing first use of awake ECMO in ARDS patients (4) (NCT01669863). In general, the exclusion criteria stressed the absence of septic or cardiogenic shock and multi-organ failure

Given are demographic and clinical characteristics at the time of ECMO initiation. Values are presented as median (25–75% interquartile range) or if categorical as numbers and percentage

BMI, Body Mass Index; HSCT, Hematopoietic Stem Cell Transplantation; AIDS, Acquired Immuno Deficiency Syndrome; CMV, Cytomegalovirus; PcP, Pneumocystis carinii Pneumonia; COPD, Chronic Obstructive Pulmonary Disease; HFNC, High Flow Nasula Cannula; NIV, Non Invasive Ventilation; FiO_2_, Fraction on inspired oxygen; PEEP, Positive End Expiratory Pressure; rpm, rounds per minute; Ppeak, Peak Pressure; SOFA, Sequential Organ Failure Assessment Score; GCS, Glasgow Coma Score; CRP, C Reactive Protein; PCT, Procalcitonin

Eleven patients (61%) required secondary intubation after a median of 4 (2–6) days. The most common cause for secondary intubation was agitation (6/11, 55%) stressing the critical role for delirium preventive strategies in these patients. The majority (4/6) of patients with agitation as primary cause for failing awake ECMO support developed agitation without any prior respiratory or circulatory deterioration. The choice of anxiolytic medication showed a trend toward a more frequent use of benzodiazepines in patients with agitation compared to all other patients (6/6 vs. 4/12), while low-dose morphine was used less frequently (2/6 vs. 7/12).

28-day-, in hospital- and 6-month mortality rates were 44% (8/18), 50% (9/18) and 50%, respectively. In-hospital mortality was 29% (2/7) in solid organ transplantation patients and 50% (3/6) in hemopoietic stem cell transplantation patients. In-hospital mortality was 73% in patients who required secondary intubation and 14% in patients who did not require intubation while on ECMO support (p = 0.023, Hazard Ratio: 0.133 (0.058–0.789)).

An exploratory analysis suggested several factor associated with later failure of an awake ECMO concept (Table 2).

**Table 2:** Predictors for failure of the awake ECMO concept

Characteristic	Secondary intubation			Logistic regression		
	No	Yes	*p*	OR	95%-CI	*p*
Benzodiazepine use during ECMO support—no (%)	2/7 (28.6)	8/11 (72.7)	0.066	6.7	0.8–55	0.078
Ppeak (NIV) before ECMO initiation—cmH2O	15 (11–16)	19 (17–22)	0.014	1.6	1–2.6	0.05
pCO2 before ECMO initiation—mmHg	37 (35–43)	50 (34–76)	0.06	1.1	1–1.2	0.163
ECMO support duration—days	9 (8–11)	12 (10–28)	0.049	1.2	0.9–1.5	0.173

Description of parameters that were associated with the necessity of later secondary intubation. Factors associated with later failure of an awake ECMO concept were more prominent use of benzodiazepines during awake ECMO support, higher peak pressures applied in noninvasive ventilation and hypercapnia directly before ECMO insertion as well as longer ECMO support. Values are presented as median (25–75% interquartile range) or if categorical as numbers and percentage

CI, Confidence Interval; NIV, Noninvasive Ventilation; Ppeak, Peak Pressure

Although this study, to the best of our knowledge, represents the largest experience with awake ECMO in ARDS patients, conclusions are still limited by its small sample size and the uncontrolled nature. Despite these limitations, our findings support the notion that an awake ECMO strategy might be a viable treatment option for immunocompromised patients with severe ARDS, especially in those patients without overt multi-organ failure. Further studies are required to determine the possible role of the awake ECMO concept in patients with ARDS.

## Data Availability

The datasets used and/or analyzed during the current study are available from the corresponding author on reasonable request.
